# Indirect transmission of tinea and the role of hair salons

**DOI:** 10.1111/jdv.70213

**Published:** 2026-01-27

**Authors:** R. J. Hay

**Affiliations:** ^1^ King's College London London UK

It has been known for many years that dermatophyte fungi infecting humans can be passed indirectly via another medium such as fomites from person to person. Initial attention focused on sporting and industrial settings and tinea pedis, where swimming baths and sporting or industry changing rooms were implicated in the transfer of infection from one individual to another. Much effort was expended trying to address this by the use of floor and shower cleaners or providing protective equipment for the users such as shower room slippers. While to some extent this addressed the problem, occupationally acquired tinea pedis was a financial burden on industries such as coal mining or nuclear fuels because of industrial injury compensation schemes [1].

The paper in the JEADV serves as a reminder that indirect transmission of dermatophytes is not restricted to shower rooms and that, in children, passing infective fungi from one to another can be accomplished through the medium of simple equipment used in hair grooming such as clippers and brushes [2]. In this Danish study of hair salons by Shen et al. 27% of brushes and 4% of hair clippers were found to be positive for pathogenic dermatophyte fungi. Similar work supports the association between hair dressing and transmission. In a study from West Africa both pathogenic fungi, including dermatophytes, as well as bacteria were found in hairdressing equipment, such as combs or shavers and scissors with a consequent risk of infection [3]. Sterilization or cleaning of these items is often carried out intermittently and not after every customer and, given the difficulty of using harmless sterilizing processes, transmission of infection remains a potential risk. The situation is exacerbated in some communities by the widespread use of neighbourhood barbers in homes that are neither subject to inspection nor are the cleaning practices as rigorous.

How much indirect transmission plays a role in other dermatophyte infections is also not so well established. The current outbreak of *Trichophyton indotineae* infection, which has spread rapidly across many different countries, is a case in point. Although suspected, this dermatophyte has not been isolated from environmental sources, apart from, occasionally, animals. Yet epidemiological surveys show a clear connection between sharing clothing or beds and the risk of infection suggesting that fomites might be a source in families for primary infection or relapse [4]. We know that the infecting elements of dermatophytes, fungal arthrospores, can survive for long periods off the human or animal host, but can be destroyed by heat above 60°C [5].

So perhaps once again we should turn our attention to considering better strategies for the prevention of tinea infections and addressing the risk from tinea capitis and, as suggested by the authors of the paper [2], hair care would be a good place to start.

## CONFLICT OF INTEREST STATEMENT

RJH has no conflicts of interest.

## Data Availability

Data sharing not applicable to this article as no datasets were generated or analysed during the current study.
